# An unanticipated cardiac arrest and unusual post-resuscitation psycho-behavioural phenomena/near death experience in a patient with pregnancy induced hypertension and twin pregnancy undergoing elective lower segment caesarean section

**DOI:** 10.4103/0019-5049.71035

**Published:** 2010

**Authors:** Mridul M Panditrao, Chanchal Singh, Minnu M Panditrao

**Affiliations:** Department of Anaesthesiology & Critical Care, Padmashree Dr. D Y Patil Medical College (Deemed University), Pimpri, Pune, India; 1 Department of Anaesthesiology, Yashoda Superspeciality Hospital, Gaziabad, UP, India

**Keywords:** LSCS, near death experience, post-CPR, pregnancy induced hypertension

## Abstract

A case report of a primigravida, who was admitted with severe pregnancy induced hypertension (BP 160/122 mmHg) and twin pregnancy, is presented here. Antihypertensive therapy was initiated. Elective LSCS under general anaesthesia was planned. After the birth of both the babies, intramyometrial injections of Carboprost and Pitocin were administered. Immediately, she suffered cardiac arrest. Cardio pulmonary resucitation (CPR) was started and within 3 minutes, she was successfully resuscitated. The patient initially showed peculiar psychological changes and with passage of time, certain psycho-behavioural patterns emerged which could be attributed to near death experiences, as described in this case report.

## INTRODUCTION

There are certain cases which remain as an enigma, in which there is always a dilemma regarding what exactly happened? What would have happened if…? So, due to any one of these reasons, they become our “Memorable Cases”.

We are presenting one such case of unusual happenings under not so unusual circumstances.

## CASE REPORT

A 24-year-old primigravida, at term with twin pregnancy, was admitted. There was no previous history of any psychiatric illness. Except for bilateral pedal oedema, a BP of 160/122 mmHg and signs of pregnancy induced hypertension (PIH), she showed no significant systemic findings. Fundoscopic examination showed normal fundal findings. Airway examination was normal. She was started on oral antihypertensives – tab. Nifedipine 10 mg, tab. Atenolol 50 mg. once a day and tab. Alpha methyldopa 500 mg twice a day, to no avail. So, after 8 days, it was decided to conduct elective LSCS under general anaesthesia. After premedication with 30 ml of 0.3 M sodium citrate orally, was brought to operating room (OR).

The patient was pre-oxygenated with 100% O_2_. Vital parameters at the time of induction were BP 154/96 mmHg, SPO_2_ 99% and heart rate 88/minute. Rapid sequence induction was done with Thiopentone (300 mg) + Succinylcholine 100 mg, applying Sellick’s maneuver during endotracheal intubation and maintained on 50% gas mixture/isoflurane (0.8–1%) with Intermittent Positive Pressure Ventilation using Vecuronium. After the delivery of both male babies, IV infusion of 20 units of Inj. Oxytocin was started. Intravenous Inj. Fentanyl 100 μg was given.

The obstetricians, after trying the manual methods for uterine contraction, were unsatisfied with “flabbiness of uterus”, and injected intramyometrialy three successive injections of 1 ml (250 mcg) of Carboprost and 1 ml (10 units) of Oxytocin each. Within 90 seconds after the injection of Carbiprost, there was associated transient bronchospasm and it was becoming little difficult to ventilate the patient, so nitrous oxide was switched off. However, within 3 minutes of the injections, the patient suffered cardiac arrest in the form of a cardiac asystole with neither peripheral nor central pulsations.

Immediately, proper drill of CPCR was executed using Inj. Adrenaline 1 ml diluted in 4 ml, given intravenously. Two successive doses were needed along with Inj. Sodium bicarbonate 50 ml IV, Inj. Atropine 0.6 mg, again as two successive doses IV. DC defibrillation was needed. Within 3 minutes, the patient was resuscitated with a heart rate of 160–180/minute, SaO_2_ gradually recovered, came to 100%, BP gradually picked up (80/60 mmHg, 100/72 mmHg and around 150/100 mmHg), EtCO_2_ was monitored and became normal. So, gradually, nitrous oxide and isoflurane were added and surgery was allowed to proceed. Blood pressure remained on the higher side (150/90 mmHg) throughout surgery. As there was neither much clinically evident blood loss nor any sign of hypovolaemia shown by the patient, blood was not transfused.

At the end of surgery, she started making spontaneous efforts; so, the extubation after adequate reversal and thorough suctioning was carried out. Patient was immediately shifted to surgical ICU.

The total duration of surgery was 85 minutes and that of GA was 100 minutes.

### Postoperative day 1

In the ICU, the patient had no recollection of antecedent events, could not recognise her relatives. She was drowsy but awake [Glasgow Coma Score (GCS) = 9]. Surprisingly, once patient was extubated and shifted to ICU, she did not show significant hypertensive response. As per the required basis, sublingual Nifedepine was administered in the ICU.

Similarly, very detailed, in-depth neurological assessment, at first with help of consultant neurologist and neurosurgeon and then psychiatric evaluation were carried out. Computed tomography (CT) scan, followed by magnetic resonance imaging (MRI) were carried out. CT scan showed only mild diffuse cerebral oedema, for which, the anti-cerebral oedema treatment in the form of 1.5 g/kg Mannitol infusion was given twice a day for 2 days. Then, MRI was done, which did not show any specific changes.

### 24 hours–1 week

The patient became more oriented but had no recall. She initially refused to accept her infants and her speech was slurred, but gradually improved with no neurological deficit and with normal vitals.

As a part of standard surgical ICU protocol, serial serum electrolytes were measured immediately postoperatively as well as later on, but did not show any abnormalities of electrolyte levels. The psychiatrists ruled out puerperal/postpartum psychosis. They did not start her on any medication and later on she did not require any treatment.

### After 1 week

She was interviewed again, this time by a psychiatrist: The consultant psychiatrists carried out the evaluation using this tool (Greyson’s NDE Scale) and were of the opinion that the score was around 8.

Peculiar findings were recorded as follows:

- She remembered going to OR, after which she was blank till about 2–3 days back when she started recognising things and relatives in ICU. In addition, she had some memories of as if “travelling in dark terrain”. She was travelling in the dark towards some bright light. She could remember people whispering that she is “dead”.
  She was discharged from hospital on 26^th^ day of admission/postoperative 18^th^ day. She was reviewed after 3 and 6 months and 1 year. She gave her history as follows.
- Whenever she was seeing herself in mirror, her sight would get focussed on the eyes of her own image and she would feel to be concentrating on the image inside the eyes of her own image.
- She also used to feel as if she was hearing many people discussing something and that someone is telling her she is no more/she is dead.
- By this time, she had accepted her offsprings and got well adjusted to marital and maternal life. At present, she is leading her normal life.

## DISCUSSION

The “near death experience” (NDE), as it is called, is a well-documented phenomenon.[1–3] Peripartum morbidity and mortality are also well-documented phenomena.

Carboprost tromethamine (Hemabate^®^),[4] a methylated analogue of prostaglandin F_2_ α (PGF_2_ α), has been in clinical practice for fairly sometime and many obstetricians use it as uteromyotonic, intramyometrially. So is it with Oxytocin too. Carboprost, Misoprostol and other uterine stimulants causing cardiac arrest have been documented.[5]

In our case, the exact cause of cardiac arrest is still a dilemma. The patient who had been anaesthetised for half an hour, suddenly going into cardiorespiratory arrest, could be due to

- delivery of foetuses (uterine incision and opening of myometrial vessels) or
- intramyometrial injections of Carboprost and Oxytocin or
- detachment of placenta and embolisation of liquor amnii through open sinuses.

The ECG, NIBP, SpO_2_ and ETCO_2_ were continuously monitored. PaO_2_ and PaCO_2_ were measured only after she suffered asystole and resuscitation was going on, which obviously were abnormal. The successful revival of the patient suggests that the event was acute, transient and not related to any organic lesion. Psychological phenomena which happened in post-CPR period have been a subject of intense evidence search and the instances of “Out Of Body” experiences or even travelling through dark tunnel towards intense light have been recorded. The experiences that our patient had can be included under the realm of NDEs, as per the Greyson’s NDE Scale (of more than 7).[6,7]

Various psychological phenomena which happen in post-CPR period have been recorded. But our patient’s few more peculiar psycho-behavioural experiences are not explainable to us. Discussion with psychiatrists has not been very conclusive. Till date, this has been an enigma to us.

In conclusion complicated obstetrics can be very challenging and can put us through unusual situations. Intramyometrial use of uterine stimulants can be very risky and should be avoided as far as possible. Making all healthcare givers well acquainted with the knowledge of “Out of Body”/NDEs in post-CPR period must be part of our curriculum. In addition to the already reported behavioural changes, new experiences could be added to the existing list.
